# The Psychometric Properties of the Arabic Preschool Activity Card Sort

**DOI:** 10.1155/2017/5180382

**Published:** 2017-03-07

**Authors:** Somaya H. Malkawi, Sana M. N. Abu-Dahab, Ahmad F. Amro, Nihad A. Almasri

**Affiliations:** ^1^Department of Occupational Therapy, The University of Jordan, Amman, Jordan; ^2^Department of Hearing and Speech, Al-Ahliyya Amman University, Amman, Jordan; ^3^Department of Physiotherapy, The University of Jordan, Amman, Jordan

## Abstract

**Background:**

The Preschool Activity Card Sort (PACS) is an interview-based assessment tool to measure participation of preschool children with age range from 3 to 6 years.

**Objective of Study:**

The purpose of this study was to establish the psychometric properties of the recently translated Arabic PACS (A-PACS).

**Methods:**

One hundred fifty-one Jordanian parents participated in the study representing different geographical areas. Children were almost equally distributed between males and females and into three age groups. Construct and concurrent validity were examined as well as the internal consistency of the scale and the test-retest reliability.

**Findings:**

The A-PACS was able to differentiate between the participation level of young and old children in the domains of education, community mobility, and low demand leisure of the A-PACS giving evidence to its construct validity and it significantly correlated with some aspects of the Vineland Adaptive Behavior Scale (VABS) giving evidence to its concurrent validity. The A-PACS showed excellent overall internal consistency (*α* = .859) for all domains and good test-retest reliability (*r* = .976, *p* < .001).

**Conclusion:**

The A-PACS can be considered as a valid and reliable tool to measure participation of preschool children with normal development from Arabic cultures. Future studies should focus on the validity of the A-PACS for use with children with disabilities.

## 1. Introduction

Participation is defined as “*involvement in life situations*” [1] according to the International Classification of Functioning, Disability, and Health model (ICF) [1]. Law [2] further defined participation as engagement in occupations of daily activities that are essential for development, life experience, and well-being. Participation is an important component in the assessment of health and functioning of children and an ultimate goal of rehabilitation in children with diverse health status and disabilities. It is especially important to assess participation of kindergarten children as their function at this age may predict their educational and social integration in later years [3].

The Preschool Activity Card Sort (PACS) is a semistructured tool to measure participation in preschool children 3–6 years of age [4]. The PACS consists of pictures of preschool children performing 85 activities categorized into seven domains: self-care, community mobility, high physical demand leisure, low physical demand leisure, social interaction, domestic, and education. The valid and reliable English version of the PACS [4] has been translated into and validated in Spanish [5] and most recently into Arabic (A-PACS; [6]) in which it was cross-culturally adapted on a Jordanian sample, yielding 95 activities categorized into the same domains as the original PACS.

The original as well as Spanish versions of the PACS were found to be valid and reliable in several studies using different groups of children. For example, the original PACS was found to be valid and reliable in terms of content and construct validity [4]. Also, the PACS was found to discriminate between preschool children with and without autism [7]. Research supports the content validity of the Spanish PACS as well [5]. Moderate concurrent validity was found between the Spanish PACS and Pediatric Evaluation of Disability Inventory (PEDI) in the self-care domains and low concurrent validity for the mobility and social domains [5]. Moreover, the Spanish PACS differentiated participation of children of recent immigrants from American preschoolers [5].

The psychometric properties of the Arabic version of the PACS are not yet studied. Therefore, the purpose of the present study is to examine the psychometric properties of the A-PACS for children aged 3 to 6 years old. Construct and concurrent validity and internal consistency and test-retest reliability were tested.

## 2. Methods

### 2.1. Participants

A total of one hundred fifty-one parents were recruited randomly responding to an advertisement placed in public areas like shopping malls, universities, community, and child care centers and in neighborhoods. Caregivers were recruited proportionally from different geographical areas of Jordan to reflect demographics obtained from the Jordanian Department of Statistics. Parents were recruited to complete the A-PACS if they had at least one child with normal development between the ages of 3 to 6 years. Moreover, parents had to speak Arabic as their native language to be included in the study. Parents were excluded if their preschool child had any physical or mental problems or had been enrolled in first grade or if Arabic was not their native language. Children were divided into three age groups; group 1 included children who are three years old up to 3 years and 11 months old, group 2 included children who are 4 years to 4 years and 11 months old, and group 3 had children who are 5 to 6 years old. Most of the participants interviewed were mothers (70.2%) with a mean age of 32.8 years. Children were almost equally distributed between males and females (52.3% males). Demographic characteristics of the participants are delineated in Table 1.

### 2.2. Measurements

In addition to the A-PACS, parents completed the Arabic version of the Vineland Adaptive Behavior Scale (VABS) [8]. The VABS is a semistructured interview administered to parents or caregivers to assess children's adaptive behavior from birth to 18 years of age. This assessment was developed and standardized for use with children with intellectual disabilities to assess their adaptive behavior in four domains and eleven subdomains: activities of daily living (domestic, personal, and community skills), communication (receptive, expressive, and written communication), socialization (interpersonal relationships, play/leisure, and coping skills), and motor (gross and fine motor skills). The VABS was chosen to investigate concurrent validity of the A-PACS because both measures assess similar constructs and are commonly used in validity studies of new tool development [9–11]. Additionally, the VABS was previously translated to Arabic [12] and permission was obtained to use the Arabic version of the translated VABS (M. Yousef, personal communication, February 21, 2015). Both English and Arabic VABS were reported to have sound psychometric properties [12, 13].

### 2.3. Procedure

Ethical Approval was obtained from the School of Rehabilitation Sciences research committee and the Deanship of Academic Research at the University of Jordan. All participants signed an informed consent after receiving detailed information about the study. Participants were informed that they had the right to withdraw from the study at any time.

A total of 151 parents participated in this study. Parents completed the A-PACS and the VABS as 1 : 1 interviews. Two trained occupational therapy researchers conducted interviews. The time to complete the two measures varied from 40 to 60 minutes. To assess test-retest reliability, a randomly selected subsample of 30 parents, 10 from each age group (3-3; 11, 4-4; 11, 5-6 years), completed the A-PACS twice in a 2-week interval.

### 2.4. Data Analysis

Statistical analyses were performed using the Statistical Package for Social Sciences (SPSS) for windows version 20.0. To establish the construct validity, a Kruskal-Wallis one-way analysis of variance by ranks was used to compare the mean subscale scores for each of the seven domains of the A-PACS in order to identify group differences. Concurrent validity was evaluated using the Pearson correlations coefficient between the A-PACS and the VABS domains and subdomains (with correlations <0.25 = little or no relationship; 0.25–0.50 = fair; 0.51–0.75 = good; >0.75 = excellent; [14]).

For establishing the A-PACS homogeneity (internal consistency), Cronbach's alpha was used with the criteria *α* ≤ 0.30 = weak; >0.50 = moderate; ≥0.80 = excellent [15]. Spearman correlation coefficient was used to calculate interitem correlations. For establishing test-retest reliability, we used *k* test and intraclass correlation coefficients (ICC) from a two-way ANOVA mixed effects model between time 1 and time 2 (after two weeks) (with ICC < 0.75 = poor to moderate and *r* ≥ 0.75 = good to excellent reliability). The same therapist collected the test-retest reliability data for the 30 participants at time 1 and time 2.

## 3. Results

### 3.1. Validity

#### 3.1.1. Construct Validity

The mean number of activities in which children participated in each of the A-PACS domains is reported in Table 2. For the education domain, participation of age group 1 was significantly lower than groups 2 and 3; however, no significant difference was found between age group 2 and age group 3. Moreover, significant differences between age group 1 and group 3 were found in community mobility and low demand leisure domains with group 3 showing higher participation than group 1. However, age group 2 was not significantly different from age group 1 or group 3 for the aforementioned domains. Finally, no significant differences between the three age groups were found for domains of self-care, high demand leisure, social interaction, and domestic activities.

#### 3.1.2. Concurrent Validity

Table 3 shows the relationships among A-PACS and VABS domains and subdomains. At the level of domains scores, significant correlations were found among the A-PACS and all the VABS. Specifically good correlations were observed with VABS communication domain (*r* = 0.629, *p* < .001), VABS social domain (*r* = 0.577, *p* < .001), and VABS motor domain (*r* = .692, *p* < .001). The only fair relationship was observed with the VABS ADL domain (*r* = 0.436, *p* = .002).

Among the domestic and social domains of the A-PACS and the VABS motor domain, fair correlations were observed (*r* = .423, *p* = .014) and (*r* = .445, *p* = .009), respectively. The A-PACS domestic domain was significantly correlated with the VABS-fine motor subdomain (*r* = .339, *p* = .050) while social domain of the A-PACS was significantly correlated with receptive communication (*r* = .315, *p* = .023), personal ADL (*r* = .335, *p* = .015), social interpersonal relations (*r* = .292, *p* = .036), gross motor (*r* = .341, *p* = .048), and fine motor (*r* = .418, *p* = .014) subdomains.

As for the low demand leisure domain of the A-PACS, it was significantly and fairly correlated with the VABS ADL (*r* = .333, *p* = .019), VABS social (*r* = .311, *p* = .029), and all of their subdomains.

The community mobility domain of the A-PACS was significantly and fairly correlated with the ADL domain of the VABS (*r* = .382, *p* = .007) and all its subdomains and the social domain of the VABS (*r* = .283, *p* = .048) and all its subdomains except for interpersonal relations subdomain, which was not significant.

Interestingly, the high demand leisure and self-care domains of the A-PACS were not significantly correlated with any of the VABS domains.

### 3.2. Reliability

#### 3.2.1. Internal Consistency

The overall internal consistency for all the 98 items of the A-PACS was excellent (*α* = .859) and its interitem correlations with A-PACS total scores were moderate in all seven domains (high demand leisure *α* = 0.559; self-care *α* = 0.568; education *α* = 0.688; community mobility *α* = 0.708; low demand leisure *α* = 0.797; domestic *α* = 0.721; and social *α* = 0.750).

#### 3.2.2. Test-Retest Reliability

The ICC (3,195) = .976 for the test-retest reliability showed good stability for the A-PACS (*F* = 40.976 with 95% confidence interval of .968 to .982, *p* < .001).

## 4. Discussion

The purpose of the current study was to establish the psychometric properties of the A-PACS that was recently translated into Arabic and cross-culturally adapted on a Jordanian population of parents of children with typical development.

In congruence with developmental theory [16], results revealed an increase in children's participation as they get older. This could be explained as child engagement in activities outside their home environment becomes more often and begins to be involved more in social play as they get older. Similar patterns were observed for children with disabilities. Law et al. [17] found that children older than 4 years participated in a broader range of activities and did so with greater intensity. Dunst and colleagues [18] found an increase in the percentage of children at risk for or with developmental delay who participated in family and community activities from birth to 6 years. Similarly, our findings indicate a higher participation level in preschool children as they grow. The findings that younger preschoolers participated in significantly lower education activities than older preschoolers are expected as kindergarten in Jordan starts at the age of 4 years. Therefore, an increase level of participation in education is noticed with older children.

Significant differences between children aged 3-4 years and children aged 5-6 years were found in community mobility and low demand leisure domains with higher participation for older children. However, there were no significant differences between children aged 3-4 years and 4-5 years. This can be explained by the nature of the items in the domains of community mobility and low demand leisure which most require exposure to new skills and experiences. For example, most of the items in the community mobility domain are taught when the children join education settings. Examples include crossing the street, taking the bus, going to a supermarket, learning money management, climbing the stairs, using a public bathroom, and going on field trips. The domain of low demand leisure includes items that require skill acquisition and tools, such as doing puzzles, using scissors, and playing with playdough. Therefore, children aged 4-5 years have just begun to learn these skills. They are emerging; the skills are not yet well established and not yet evident to parents. However, when children enter their second level of kindergarten after they have had an entire of exposure to these experiences, parents are able to see the difference and report it.

When comparing the 3 age groups, the overall pattern of participation appeared similar in the domains of self-care, high demand leisure, social interaction, and domestic activities. This result can be attributed to the nature of the items in aforementioned domains as these activities mostly do not require specific tools or skills such as jumping and running. Moreover, for the domestic domain, it is noteworthy to notice that Jordanian children reported low participation level at all age groups as noted in Table 2. Domestic activities such as “cooking, cleaning, sweeping, and taking the trash out” did not differ among the three groups simply because most of the Jordanian children are not usually engaged in instrumental activities of daily living (IADL) at this age stage. These concepts are introduced in general education at the level of grade 1 and above and thus are practiced at that age at home.

The Arabic version of the VABS was used in this study to explore the concurrent validity of the A-PACS. Results of this study suggest that some domains of the A-PACS correlated with others in the VABS. However, most of these correlations were fair.

Only the education domain of the A-PACS correlated with all of the VABS domains and subdomains. An important observation is that, with the exposure of children to educational experiences in kindergarten, children are getting training in all of the other domains like communication, ADL, social, and motor skills. A lot of preschoolers form new repertoire of social interaction and communication, they learn social behaviors and morals, and they get to go on field trips to parks and play areas which will increase their motor skills. They get exposed to academics and use pencils and utensils and they are encouraged to do self-care activities like brushing their teeth. Consequently, as participation in the number of educational activities as measured by the A-PACS increased, an increase in participation level in all domains and subdomains of the VABS was noted.

The number of significant correlations between the A-PACS and the VABS differed depending on the domain; education domain of the A-PACS had the highest and strongest correlations (15 correlations) which indicates that education might have a positive impact on child's skills. Based on the number of correlations, the low demand leisure domain ranked second (11 correlations), followed by the community mobility and social domain (6 correlations each) and the domestic domain (2 correlations).

An interesting finding was that the A-PACS low demand leisure was fairly and significantly correlated with the play and leisure subdomain (social-VABS) and the A-PACS social domain was significantly and fairly correlated with the interpersonal relations (social-VABS) which indicates that the two tests are examining different activities of the same domain suggesting that both tests complement each other.

The finding that no correlation existed between the self-care and high demand leisure domains of the A-PACS and any of the domains or subdomains of the VABS might be explained by the way items are distributed into domains in each of the A-PACS and the VABS. We found that items in the A-PACS were sporadically distributed in different domains of the VABS which made it increasingly difficult to make obvious correlations between one domain and the other. For example, the self-care items of the A-PACS are similar to items on the VABS that are found across domains and not specifically situated in the personal living skills section alone. In other words, the self-care and high demand leisure domains of the A-PACS bring added evaluative value and information than using the VABS clinically.

The excellent internal consistency for all the 98 items of the A-PACS reflects the appropriateness of the new items added to the original tool in measuring participation levels. However, the relatively lower value of Cronbach's alpha for the high demand leisure (0.559) may reflect variability in frequency of participation across children in the study sample.

The ICC coefficients between the first and second A-PACS scores varied from .96 to .98 supporting test-retest reliability. This finding indicates that when change is not expected scores are sufficiently stable to support using the A-PACS to measure change over time.

## 5. Limitations and Recommendations

Small sample size in this study limits the generalizability of the results; therefore, results should be interpreted with caution. Further studies should be completed with larger sample sizes. The validity of the A-PACS for use with children with disabilities should also be explored. Finally, further studies are needed to test whether the A-PACS is sensitive to change in children. In conclusion, the A-PACS have demonstrated acceptable psychometric properties. Therefore, the A-PACS can be used as a reliable and valid measure to assess children's participation at the age of 3–6 years who speaks the Arabic language.

## Figures and Tables

**Table 1:** Demographic characteristics of the sample.

	Total *N* = 151 *f* (%)	Age groups		
		Group 1 (3-3, 11) *n* = 57 *f* (%)	Group 2 (4-4, 11) *n* = 42 *f *(%)	Group 3 (5-6) *n* = 52 *f*(%)
Parent				
Mother	106 (70.2)	41 (38.7)	28 (26.4)	37 (34.9)
Father	45 (52.3)	16 (35.6)	14 (31.3)	15 (33.3)
Gender of child				
Male	79 (52.3)	30 (52.6)	19 (45.2)	30 (57.7)
Female	72 (47.7)	27 (47.4)	23 (54.8)	22 (42.3)
Socioeconomic status				
1	52 (35.4)	20 (36.4)	15 (37.5)	17 (32.7)
2	65 (44.2)	20 (36.4)	20 (50.0)	25 (48.1)
3	20 (13.6)	11 (20.0)	5 (12.5)	4 (7.7)
4	7 (4.8)	3 (5.5)	0 (0.0)	4 (7.7)
5	3 (2.0)	1 (1.8)	0 (0.0)	2 (3.8)
Living area				
City	107 (72.3)	39 (68.4)	30 (76.9)	38 (73.1)
Village	41 (27.7)	18 (31.6)	9 (23.1)	14 (26.9)
Mother's age (years), M (SD)	32.8 (5.7)	32.2 (5.7)	31.8 (4.9)	34.1 (6.1)
Father's age (years), M (SD)	37.9 (5.8)	37.5 (5.8)	37.1 (4.8)	39.0 (6.3)

**Table 2:** Mean number of A-PACS activities reported by total sample and age group.

A-PACS	Total sample	Group 1 *N* = 57 3-3, 11 M [CI]	Group 2 *N* = 42 4-4, 11 M [CI]	Group 3 *N* = 52 5-6 M [CI]
Self-care	15.35	15.29^a^ [14.89,15.70]	15.30^a^ [14.87,15.78]	15.10^a^ [15.10,15.73]
Community mobility	13.08	12.38^a^ [11.87,12.89]	13.28^ab^ [12.77,13.79]	13.69^b^ [13.17,14.20]
HD leisure	8.19	8.05^a^ [7.71,8.38]	8.16^a^ [7.81,8.51]	8.36^a^ [7.93,8.79]
LD leisure	15.20	14.70^a^ [14.18,15.21]	15.04^ab^ [14.38,15.71]	15.88^b^ [15.32,16.44]
Social interaction	13.36	13.03^a^ [12.54,13.52]	13.45^a^ [13.02,13.87]	13.65^a^ [13.16,14.14]
Domestic	5.11	4.61^a^ [4.08,5.13]	5.35^a^ [4.77,5.93]	5.48^a^ [4.90,6.05]
Education	9.09	8.21^a^ [7.68,8.74]	9.42^b^ [8.88,9.97]	9.80^b^ [9.28,10.33]

Note: a, b, and c = if letters are the same, there are no significant differences between groups.

A-PACS = Arabic Preschool Activity Card Sort; HD = high demand; LD = low demand.

**Table 3:** Relationships among A-PACS and Vineland domains and subdomains for total sample.

Vineland	A-PACS						
	*Self-care*	*CM*	*HD leisure*	*LD leisure*	*Social*	*Domestic*	*Education*
	*r* *p* *n*	*r* *p* *n*	*r* *p* *n*	*r* *p* *n*	*r* *p* *n*	*r* *p* *n*	*r* *p* *n*
*Communication*	.155 .287 49	.153 .294 49	.002 .990 49	.169 .245 49	.229 .113 49	.096 .513 49	.629^*∗∗*^ .000 49
Receptive	.070 .622	.221 .115	.211 .134	.371^*∗∗*^ .007	.315^*∗*^ .023	.055 .698	.453^*∗∗*^ .001
Expressive	.109 .442	.196 .164	.097 .493	.211 .134	.250 .074	.081 .569	.686^*∗∗*^ .000
Written	.194 .169	.193 .325	.051 .719	.201 .154	.148 .295	−.095 .501	.450^*∗∗*^ .001

*ADL*	.101 .488 49	.382^*∗∗*^ .007 49	.086 .557 49	.333^*∗*^ .019 49	.209 .150 49	.152 .298 49	.436^*∗∗*^ .002 49
Personal	.176 .211	.438^*∗∗*^ .001	.201 .154	.436^*∗∗*^ .001	.335^*∗*^ .015	.255 .068	.553^*∗∗*^ .000
Domestic	.138 .330	.407^*∗∗*^ .003	.110 .438	.345^*∗*^ .012	.167 .236	.171 .225	.441^*∗∗*^ .001
Community	.194 .169	.326^*∗*^ .018	.041 .773	.402^*∗∗*^ .003	.226 .107	.105 .458	.469^*∗∗*^ .000

*Social*	.095- .517 49	.283^*∗*^ .048 49	.087 .550 49	.311^*∗*^ .029 49	.237 .100 49	.165 .256 49	.577^*∗∗*^ .000 49
Interpersonal relations	.006 .967	.167 .237	.137 .333	.324^*∗∗*^ .019	.292^*∗*^ .036	.128 .367	.491^*∗∗*^ .000
Play and leisure	−.044 .759	.370^*∗∗*^ .007	.152 .283	.380^*∗∗*^ .006	.218 .120	.109 .433	.514^*∗∗*^ .000
Coping	−.086 .545	.355^*∗∗*^ .010	.063 .659	.286^*∗*^ .040	.210 .136	.100 .481	.567^*∗∗*^ .000

*Motor*	.191 .287 33	.146 .417 33	.294 .097 33	.427^*∗*^ .013 33	.445^*∗*^ .009 33	.423^*∗*^ .014 33	.692^*∗∗*^ .000 33
Gross	−.035 .845	.098 .583	.314 .071	.281 .107	.341^*∗*^ .048	.220 .211	.643^*∗∗*^ .000
Fine	.069 .699	.298 .087	.276 .115	.338 .051	.418^*∗*^ .014	.339 .050	.709^*∗∗*^ .000

Note: ^*∗*^*p* < .05; ^*∗∗*^*p* < .01.

A-PACS = Arabic Preschool Activity Card Sort; CM = community mobility; HD = high demand; LD = low demand.
